# Laparoscopic conversion of omega loop gastric bypass to Roux-en-Y gastric bypass for Barrett’s esophagus: case report

**DOI:** 10.1186/s12893-022-01695-9

**Published:** 2022-07-14

**Authors:** U. G. Lange, Y. Moulla, M. Mehdorn, J. Tuennemann, A. Zabel-Langhennig, A. Ouaid, A. Dietrich

**Affiliations:** 1 Clinic for Visceral, Transplant and Thoracic and Vascular Surgery, University Hospital Leipzig, Liebigstrasse 20, 04103 Leipzig, Germany; 2 Division of Gastroenterology, Department of Internal Medicine, Neurology and Dermatology, University Hospital of Leipzig, Leipzig, Germany; 3 Division of Gastroenterology, Department of Internal Medicine II, St. Elisabeth Hospital, Leipzig, Germany

**Keywords:** Barrett’s metaplasia after MGB-OAGB, Case report, Bile reflux, Complications MGB-OAGB, Conversion bariatric procedures

## Abstract

**Background:**

The number of mini gastric bypass / one anastomosis bypass (MGB-OAGB) procedures in bariatric patients that have been performed world-wide has drastically increased during the past decade. Nevertheless, due to the risk of subsequent biliary reflux and development of ulcer and neoplastic (pre)lesions caused by long-time bile exposure, the procedure is still controversially discussed. In here presented case report, we could endoscopically demonstrate a transformation from reflux oesophagitis to Barrett’s metaplasia most likely caused by bile reflux after mini-gastric bypass. To our knowledge, this is a first case study that shows development of Barrett’s metaplasia after MGB-OAGB.

**Case presentation:**

We present the case of a 50-year-old female which received a mini-gastric bypass due to morbid obesity (body mass index (BMI) 42.4 kg/m^2^). Because of history gastroesophageal reflux disease (GERD), a fundoplication had been performed earlier. Preoperative gastroscopy showed reflux esophagitis (Los Angeles classification grade B) with no signs of Barrett’s metaplasia. Three months post mini-gastric bypass, the patient complained about severe bile reflux under 40 mg pantoprazole daily. Six months postoperative, Endoscopically Barrett’s epithelium was detected and histopathologically confirmed (C1M0 after Prague classification). A conversion into Roux-en-Y gastric bypass was performed. The postoperative course was without complications. In a follow up after 6 months the patient denied reflux and showed no signs of malnutrition.

**Conclusions:**

The rapid progress from inflammatory changes of the distal esophagus towards Barrett’s metaplasia under bile reflux in our case is most likely a result of previous reflux disease. Nevertheless, bile reflux appears to be a potential decisive factor. Study results regarding presence of bile reflux or development of endoscopically de-novo findings after MGB-OAGB are widely non-conclusive. Long-term prospective studies with regular endoscopic surveillance independent of clinical symptoms are needed.

## Background

Since the implementation of mini-gastric bypass in 1997 by Rutledge, the procedure showed a steady increase and is meanwhile the third most popular bariatric operation after sleeve gastrectomy and Roux-Y gastric bypass [1]. Following positive short- and mid-term follow-up reports [2, 3] and first randomized-trials [4], the International Federation for the Surgery of Obesity and Metabolic Disorders (IFSO) recognized mini gastric bypass / one anastomosis bypass (MGB-OAGB) as effective and safe procedure. In 2018, IFSO has recommended MGB-OAGB for surgical treatment of obesity and metabolic diseases [5]. MGB shows good and comparable results concerning postoperative complications, weight loss and improvement of type 2 diabetes, However, one aspect of MGB is controversially discussed: the theoretically risk of post-procedure biliary reflux and development of ulcer and neoplastic (pre)lesions.

The frequency and value of bile reflux (after MGB or OAGB) has been discussed with some controversy. The group of Keleidari et al*.* detected no significant difference in bile reflux endoscopically between Roux-en-Y gastric bypass (RYGB) and OAGB (4.7% vs. 1.7%) in their trial with 122 patients [6]. On the other hand, the YOMEGA-Trial reported 15.5% of cases with bile reflux in the gastric pouch versus none in the RYGB collective (randomized, controlled trial with 234 patients) [7]. These latter findings are supported by the interim results of the RYSA Trial with 40 patients undergoing OAGB. Bile reflux here was a common finding 6 months postoperatively (28.9% with bile in gastric pouch and 2.6% in the oesophagus) using bile reflux scintigraphy [8].

The sequence “reflux disease—Barrett’s metaplasia—adenocarcinoma” has been well studied. The animal model of Sato et al*.* showed the effect of duodeno-esophageal reflux in gastrectomized rats, leading to Barrett’s epithelium within 10 weeks, to dysplasia and adenocarcinoma after 20 weeks [9]. Similar results were obtained by Bruzzi et al., who reported an increased bile acid concentration in the esogastric segment of OAGB rats compared to sham. The authors found a mean bile acid concentration 2.8 times higher in the OAGB group at 7 weeks, that increased to 4.2 times higher at 16 weeks follow-up. On gastric cardia biopsies, they reported a significant increase of eosinophilic polynuclear cell infiltration into the chorion of OAGB rats, but no intestinal metaplasia [10]. Also, other human-based studies characterize bile reflux as strong major player in development and progression of Barrett’s esophagus [11–14]. There are a few case reports about conversion of MGB-OAGB to RYGB because of symptomatic bile reflux [15, 16] and there are two cases, where 2 years after MGB an adenocarcionoma of the gastro-esophageal junction has developed [17, 18]. However, long-term studies that thoroughly evaluate a (pre)carcinogenic effect of bile reflux are lacking.

## Case presentation

In May 2018, a 50-year-old female with the diagnosis of morbid obesity (body mass index (BMI) 42.4 kg/m^2^ presented to our centre. Secondary illnesses were diabetes mellitus type II (non-insulin-dependent), gastroesophageal reflux disease (GERD), hypothyroidism and oligoarthritis. A laparoscopic fundoplication after Nissen-Rossetti with mesh repair of a hiatus hernia (Bio A mesh, Gore®) had been performed for GERD in spring 2014 (patient BMI was 30 kg/m^2^). Due to recurrent reflux disease, an open refundoplication with gastropexy and simultaneous cholecystectomy had been performed in 2016. Both operations have been realized in an external hospital.

**Fig. 1:**
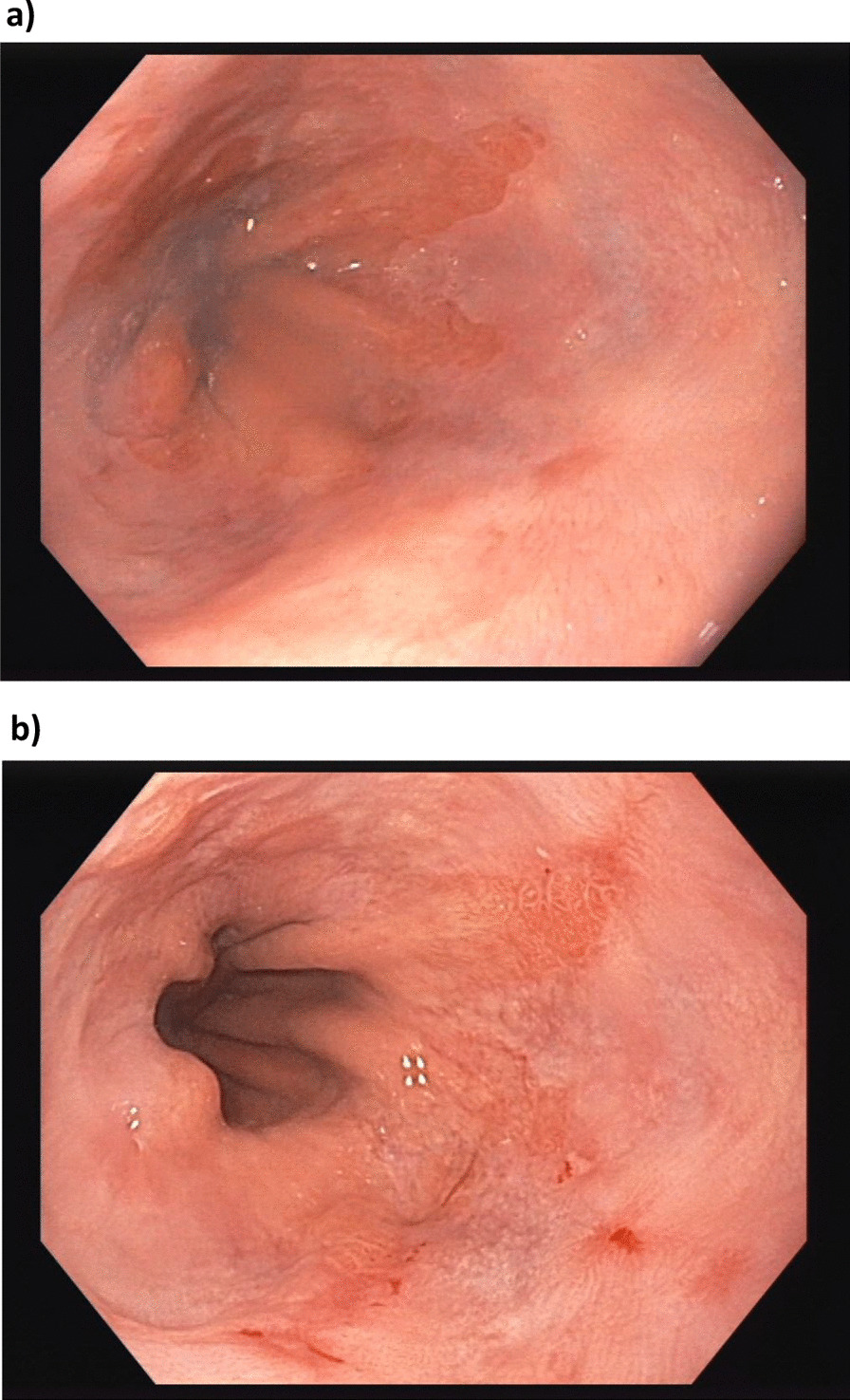
**a **Reflux esophagitis, no Barrett epithelium 09/2016. **b** Reflux esophagitis 05/2018

Biopsies of the esophagogastric junction taken in a control gastroscopy from 09/2016 showed inflammatory changes grade II after Elster, no Barrett epithelium (see Fig. 1a). Additionally, a hiatal hernia was detected. A gastroscopic control from May 2018 due to clinical symptoms of reflux confirmed a reflux esophagitis (Los Angeles (LA) classification grade B) (see Fig. 1b).

Because of distinct immobility and little prospect of improvement with conservative therapy, the decision for metabolic surgery was taken and confirmed after discussion in our obesity board. It was decided to perform a laparoscopic Roux-en-Y gastric bypass due to persistent reflux disease despite a daily dose of pantoprazole 40 mg and the procedure was scheduled as for December 2018.

Intraoperatively, the hiatus was exposed after adhesiolysis of adhesions of the omentum and transverse colon to the abdominal wall and liver. No hiatal hernia was observed. The fundoplication was reversed. Due to adhesions and scary tissue of the proximal stomach, it was decided not to create a small pouch as suitable for Roux-en-Y gastric bypass. Instead, we undertook an Omega-Loop gastric bypass,with a native gastric wall for pouch creation and gastroenteroanastomosis in the mid and distal stomach. A slim gastric pouch was created along a floppy 28fr bougie around 15–18 cm in length. Treitz ligament was identified and the biliopancreatic limb was measured 200 cm. Gastrojejunostomy was performed as a vertical isoperistaltic, antecolic, side-to-side 40 mm-gastrojejunostomy at the posterior pouch wall. The postoperative course was without complications.

**Fig. 2:**
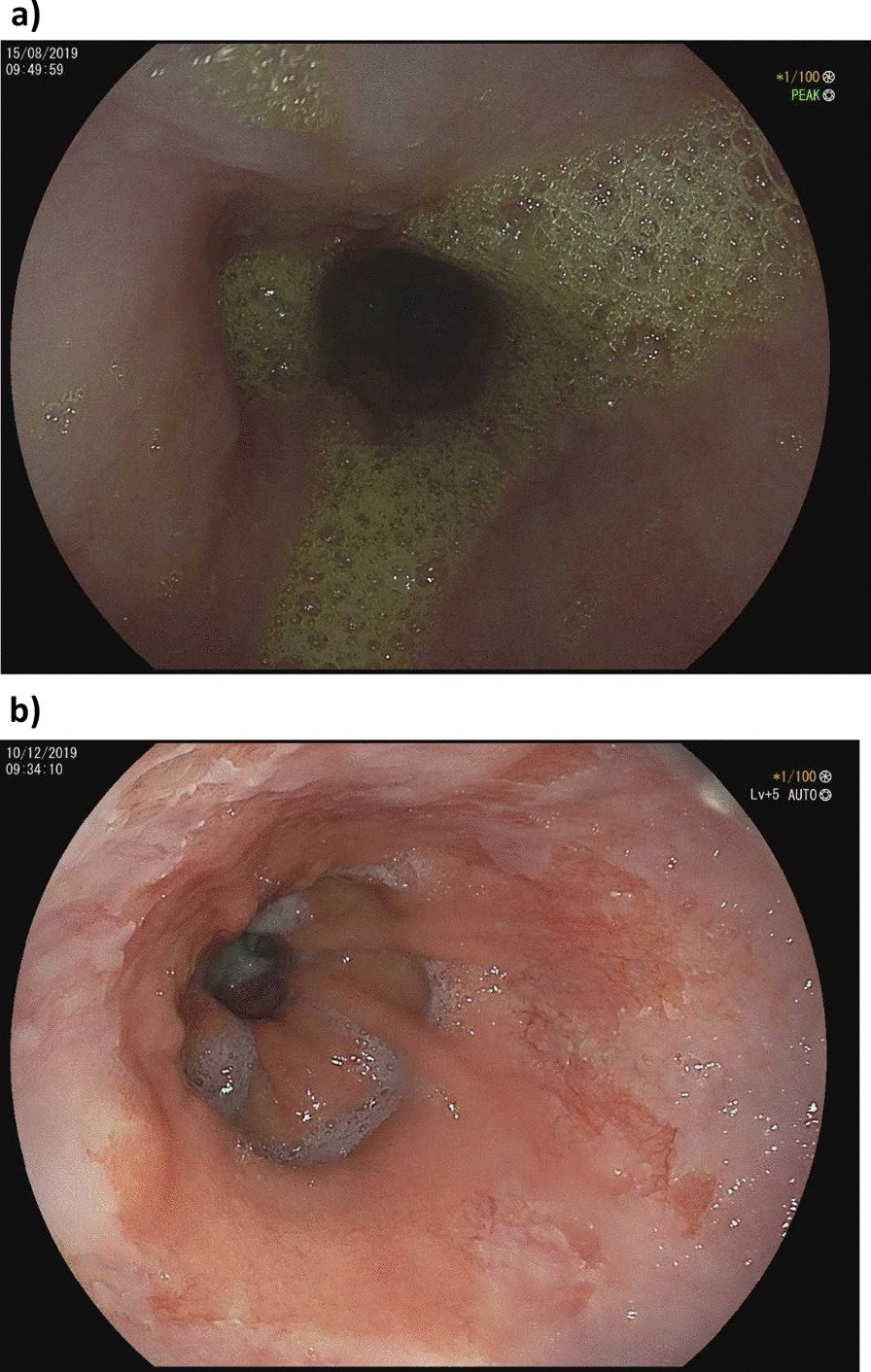
**a **Bile reflux in the esophagus 08/2019. **b** Barrett’s esophagus 12/2019

At the 3 months follow up visit, the patient already complained about severe (bile) reflux despite 40 mg pantoprazole daily. In August 2019, bile reflux in the esophagus was confirmed by gastroscopy and a reflux esophagitis was detected (grade A after LA classification) (see Fig. 2a). Biopsies of the distal esophagus showed Barrett’s epithelium without intraepithelial neoplasia. An endoscopic control 6 months later confirmed Barrett’s oesophagus (C1M0 according to Prague classification), histopathological persistent without intraepithelial neoplasia (see Fig. 2b).

We therefore set the indication for conversion into a Roux-en-Y gastric bypass due Barrett’s esophagus in our obesity board. The BMI at this time was 31 kg/m^2^. The procedure was performed successfully and without complications in spring 2020 with a length of the biliopancreatic limb of 80 cm and the alimentary limb of 150 cm (the previous 200 cm biliopancreatic limb was now measured 150 cm). The postoperative course again was without complications; the patient was discharged 4 days after surgery.

In a follow up after 6 months the patient denied reflux and showed no signs of malnutrition. A regular endoscopic control because of Barrett’s metaplasia will now be implemented.

## Discussion and conclusions

In here presented case we could endoscopically demonstrate a transformation from reflux oesophagitis to Barrett’s metaplasia, most likely caused by bile reflux after mini-gastric bypass. These findings resulted in a conversion to RYGB. As far as we are aware, this is the first case report published on development of Barrett’s metaplasia after MGB-OAGB.

The fear of carcinoma through bile reflux is historically reasoned and has been controversially discussed since implementation of MGB-OAGB. Li et al*.* reported a high incidence of esophagus adenocarcinoma in patients that underwent partial gastrectomy, a condition similar to OAGB due to changed reflux components—more bile—rather than gastric acid reflux after gastrectomy [19]. However, the reported percentages of bile reflux after MGB-OAGB show a wide range and differ from 0.3 to 36.6% [3, 8, 20–24]

Notably, de novo endoscopical findings at the gastro-esophageal junction and esophagus after MGB-OAGB have been reported. A recent study of Pizza et al*.* showed endoscopically significant more Grade A-B esophagitis after OAGB than RYGB in the 1 year follow-up. However, no cases of Barrett’s metaplasia were detected detected [25]. Also, the YOMEGA-trial found significantly more histopathologically-proven esophagitis (especially grade A-B) 2 years after OAGB compared to RYGB [7]. In a study by Saarinen et al., de novo findings 6 months postoperatively included 15.8% of inflammation at the gastro-esophageal junction, 7.9% of gastric metaplasia and 15.8% of esophagitis [8].

The group of Pizza et al*.* [26] discussed two interesting points: firstly, they noted that bariatric patients are often adapted to reflux symptoms and therefore the clinically reported numbers due to reflux scoring systems do not correspond to the endoscopic findings. Secondly, the intake of proton pump inhibitors is practiced more often and longer than recommended, leading to a potential cover-up of GERD symptoms. Therefore, a long-term prospective study with regular endoscopic surveillance independent of clinical symptoms is needed to record changes objectively.

Furthermore, the duration of bile exposure is a main determinant in the pathogenesis of complications related to biliary reflux. Intestinal metaplasia may take several years to develop. Bruzzi et al*.* could detect significant histopathological changes after 4-months in their gastric cardia biopsies, but no intestinal metaplasia. The authors concluded that the 4-month evaluation in rats represents an equivalent exposure to biliary reflux of 12 to 16 years of human life, and this may be insufficient when analysing carcinogenesis risk. Again, trials with a long-term follow-up are needed to make reliable statements about the carcinogenic risk of bile reflux in the esophagus after MGB-OAGB.

The rapid progress from inflammatory changes of the distal esophagus to Barrett’s metaplasia under bile reflux in our case is most likely a result of previous reflux disease. in this regard, it must be pointed out that the previous operations for reflux treatment (fundoplicatio and re-fundoplicatio) generally do not show good long-term results in patients with obesity. For example, Schietroma et al. [26] demonstrated that laparoscopic fundoplicatio after Nissen-Rossetti significantly increased the incidence of esophagitis, relapse and required reoperation. An RYGB, on the other hand, would be the recommended procedure according to the study group. Unfortunately, we did not perform functional diagnostics (manometry, pH-metry) in advance to our initial bariatric surgery (also this would also have provided a good postoperative comparison). However, this is especially useful in patients with significant symptoms of gastroesophageal reflux may influence the surgical technique [27, 28]. Certainly, in our case, the oesophageal mucosa was significantly damaged by the untreated reflux and transformation to Barrett's mucosa was likely, yet the endoscopically proven bile reflux after mini-gastric bypass seems to have been a determining factor regarding rapid deterioration.

## Data Availability

Not applicable.

## Abbreviations

BMI: Body mass index
LA classification: Los Angeles classification
MGB-OABG: Mini gastric bypass-one anastomosis gastric bypass
RYGB: Roux-en-Y gastric bypass
